# Integrated health and care systems in England: can they help prevent disease?

**DOI:** 10.1136/ihj-2019-000013

**Published:** 2020-02-26

**Authors:** Adam D M Briggs, Anya Göpfert, Ruth Thorlby, Dominique Allwood, Hugh Alderwick

**Affiliations:** 1 The Health Foundation, London, UK; 2 Warwick Medical School, University of Warwick, Coventry, West Midlands, UK

**Keywords:** health policy, qualitative research, health services research

## Abstract

**Objectives:**

Over the past 12 months, there has been increasing policy rhetoric regarding the role of the National Health Service (NHS) in preventing disease and improving population health. In particular, the NHS Long Term Plan sees integrated care systems (ICSs) and sustainability and transformation partnerships (STPs) as routes to improving disease prevention. Here, we place current NHS England integrated care plans in their historical context and review evidence on the relationship between integrated care and prevention. We ask how the NHS Long Term Plan may help prevent disease and explore the role of the 2019 ICS and STP plans in delivering this change.

**Methods:**

We reviewed the evidence underlying the relationship between integrated care and disease prevention, and analysed 2016 STP plans for content relating to disease prevention and population health.

**Results:**

The evidence of more integrated care leading to better disease prevention is weak. Although nearly all 2016 STP plans included a prevention or population health strategy, fewer than half specified how they will work with local government public health teams, and there was incomplete coverage across plans about how they would meet NHS England prevention priorities. Plans broadly focused on individual-level approaches to disease prevention, with few describing interventions addressing social determinants of health.

**Conclusions:**

For ICSs and STPs to meaningfully prevent disease and improve population health, they need to look beyond their 2016 plans and fill the gaps in the Long Term Plan on social determinants.

**Strengths and limitations of this study**

**What is already known about this subject?**

- The National Health Service (NHS) in England plans for the entire country to be covered by integrated care systems (ICSs) by April 2021. The aims of these local health and care partnerships are broad and include improving disease prevention and population health while maintaining NHS financial sustainability. Yet, the evidence for more integrated care leading to better disease prevention is weak.

**What does this study add?**

- Although nearly all of the 2016 sustainability and transformation partnership (STP) plans included a prevention or population health strategy, the content varied widely, often lacked detail, and had little on population-level interventions affecting the social determinants of health.

**How might this impact on clinical practice or future developments?**

- The 2019 STP and ICS 5-year strategic plans, and the roll out of ICSs across England by April 2021, provide an opportunity for local health and care services to work together more effectively to prevent disease and improve population health.
- In light of limited evidence on the relationship between integrated care and disease prevention, we describe how a more coordinated approach to tackling the social determinants of health across government could help.

The idea that the National Health Service (NHS) should focus on preventing disease as well as treating it is nothing new, but the policy rhetoric regarding its role has been amplified over the past year. In November 2018, the Secretary of State for Health and Social Care published a policy paper describing the UK Government’s ‘vision’ for prevention, which states that ‘the NHS and local authorities need to put prevention at the heart of everything they do’.^1^ This was followed in January 2019 with the launch of the NHS Long Term Plan—a blueprint for how the NHS in England will spend additional government funding over the coming 5 years—which, along with the subsequent implementation framework, emphasises the NHS’s role in prevention and reducing inequalities.^2 3^ Finally, the government’s recent green paper (a policy paper for consultation) echoes the commitments of the NHS Long Term Plan.^4^

These reports argue that little will be gained in disease prevention without closer working between NHS and non-NHS organisations, and without greater attention paid to social determinants of health, such as housing, education, and employment. Despite this, local governments in England who are responsible for spending on services that affect many of these social determinants faced a £3.2 billion funding gap in 2019/2020, with their money dedicated to improving health and reducing inequalities—the public health grant—being 25% less per person than in 2014/2015.^5 6^ More recent promises suggest that such cuts may start to be reversed^7^; however, the scale and timing of funding increases remain uncertain.

NHS England has suggested that one route to improved health and disease prevention is through developing ‘place-based’ health and care systems across England, called integrated care systems (ICSs). ICSs are local partnerships between NHS organisations, local government, and other relevant groups, asked to take joint responsibility for the health and care of populations of around one to three million people. They are expected to evolve from similar collaborations called sustainability and transformation partnerships (STPs) that have been in place since 2016. Although the aims of STPs and ICSs are broad, NHS England hopes that they can boost disease prevention and improve population health while maintaining NHS financial sustainability, and asked for ICSs and STPs to agree their 5-year plans for achieving these and other priorities by mid-November 2019, with publication expected in 2020.^2 3 8 9^

Here, we place current NHS integrated care plans in their historical context and review evidence on the relationship between integrated care and prevention. We ask how the NHS Long Term Plan may help improve disease prevention and—drawing on our analysis of the 2016 STP plans—explore the role of the 2019 ICS and STP plans.

## Integrated care in England

Integrated care means different things to different people and has taken various forms in English health policy.^10^

**Figure 1:**
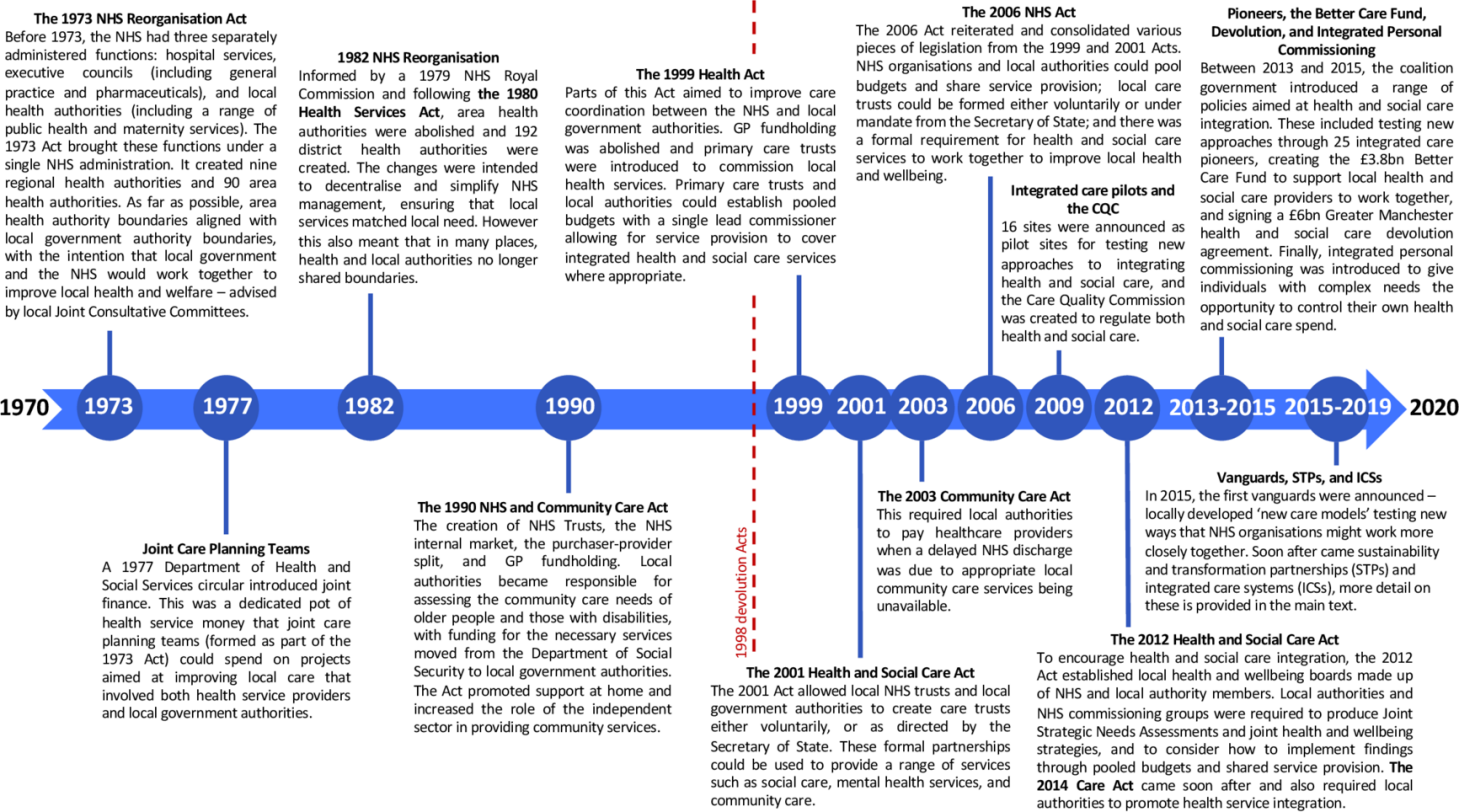
Timeline of National Health Service integrated care reforms.^12–14^

One example is more closely integrated services, particularly between NHS and social care.^11^ Since the NHS was formed in 1948, responsibility for providing social care—long-term support services for ongoing care needs resulting from old age, poverty, illness, or disability—has rested with local government rather than being coordinated nationally. This separation of authority has led to stark differences in how health and social care services are provided and funded, and to stubborn obstacles to their integration. The government has made repeated attempts to overcome these obstacles, either by incentivising or supporting health and care organisations to work together when designing and implementing services, recognising that a complex and fragmented system is difficult for patients to navigate and may lead to competing organisational incentives misaligned with improving population health (figure 1).^11–14^

Integrated care can also refer to organisations working more closely with one another. Examples include NHS organisations coordinating services between one another, and health and social care organisations aligning governance structures, budgets, and planning approaches (often in the hope of delivering more integrated services). The 2012 Health and Social Care Act, the 2014 Care Act, and the 2014 NHS Five Year Forward View (a plan for how the NHS in England intended meet patient needs through to 2020/2021) introduced a range of initiatives aimed at closer integration both between organisations providing NHS services, such as between GPs and acute hospital trusts, and between NHS and social care providers.^15–17^ To help implement the Five Year Forward View, NHS England divided the country into 44 areas covering populations between 300 000 and 3 million and asked local NHS organisations to work with local governments within each area to produce sustainability and transformation plans (with the member organisations subsequently branded STPs).^18^ These 44 plans were intended to outline how each STP would improve local population health and well-being, service quality, and healthcare efficiency between 2016 and 2021.^19^

Bringing the narrative to the present day, the 2019 NHS Long Term Plan proposed that by April 2021, ICSs will cover the entire country. In general terms, ICSs are—like STPs—local health and care systems responsible for planning and providing care for a population of between 1 and 3 million. They compose of partnerships between NHS and local government organisations, and have a partnership board and independent chair. In reality, they are likely to be heterogeneous in size, governance, and organisational composition and history. For example, three recently created ICSs cover populations ranging between 1.8 million and 3.1 million, and two of the three include at least one smaller pre-existing ICS.^20^ NHS England suggests the ICSs will have greater autonomy and responsibility than STPs for resource management, delivering standards, and improving population health.^3 21 22^ For example, they may have more freedom than STPs to choose how they spend their money but will be held to account for the overall health of their local population (such as disease prevalence or morbidity) and for addressing local health inequalities. To help achieve these aims, ICSs are expected to ‘provide stronger foundations for working with local government and voluntary sector partners on the broader agenda of prevention and health inequalities’,^2^ although it should be noted that a close partnership with local government is not guaranteed. Indeed, the original STPs were criticised for not involving local government closely enough.^19 23^

## Does more integrated care help prevent disease?

Evidence of the relationship between either service integration or organisational integration and disease prevention is limited. While studies have often explored the impact of integrated care on service use, reporting primary or secondary disease prevention activities or outcomes (stopping a disease from occurring or early identification of disease to limit its impact) is far less common.^24^

As a result, policies aimed at integrating services such as the Better Care Fund (set up in 2015 in England to help health and social care providers to pool budgets and coordinate services) and the new care models programme (introduced following the Five Year Forward View to encourage closer working between NHS services) have been evaluated based on service use rather than population health outcomes or disease prevention more broadly.^25 26^ Evaluations of more closely integrating health and care organisations have generally explored the barriers and facilitators to partnership formation rather than their impact on disease prevention or improving health. Examples include ongoing evaluations of Greater Manchester’s devolved health and social care budget (although a quantitative evaluation, including wider health impacts, is also ongoing),^27^ and studies investigating the 25 sites around England chosen to be health and social care integrated care pioneers in 2013.^28^

Policy makers in other countries have also made significant efforts to get healthcare organisations and services to work more closely. The USA, for example, has created accountable care organisations (ACOs)—groups of provider healthcare organisations financially incentivised to work together to reduce the overall costs of care they provide to an attributed population while maintaining quality of care. They were first trialled following the introduction of the Affordable Care Act, and there is a growing body of literature evaluating their effect on service use, care quality and financial savings.^29^ However, only a handful of studies have explored the impact of ACOs on preventive care, so far focusing on the use of disease prevention services such as vaccinations and screening, with mixed findings.^24 30–35^ Some ACOs are now seeking to prevent disease through addressing patients’ social needs, such as supporting access to housing, transportation and food, either directly or via community partnerships.^36^ More generally, evaluations of partnership working between organisations providing health and social services have found little evidence of health benefits.^37 38^

## Can the NHS Long Term Plan help?

The Long Term Plan states that the long-standing aim of the NHS in England is to ‘prevent as much illness as possible’.^2^ The Plan frames prevention in the context of reducing the burden of unhealthy behaviours and tackling social determinants of health (primary prevention) and improving early disease identification (secondary prevention).

However, it is unclear whether the Plan’s proposals will measurably prevent disease across England. Chapter 2 focuses on what the NHS can do to reduce harm from specific risk factors such as smoking, obesity, and alcohol through evidence-based interventions like the Ottawa Model for Smoking Cessation and scaling up the NHS Diabetes Prevention Programme.^39 40^ Yet—as the Plan points out—the main determinants of ill health lie beyond the reach of the NHS and are instead due to our social, economic, and environmental circumstances.^41^ The Plan hopes to influence some of these social determinants through training social prescribing ‘link workers’ to connect patients with relevant community services, such has housing support or weight management services, and by partnering with local charities and social enterprises. Despite being well intended, the impact of these efforts alone is likely to be limited: the evidence base on the effectiveness of social prescribing improving health is weak,^42^ social prescribing is reliant on the presence of relevant local services to address people’s social needs that have been significantly impacted by ongoing cuts to local government and other services,^43 44^ and the suggested proposals do little to address the underlying societal causes of ill-health such as employment, housing, and economic security.

The Plan also suggests some governance and payment system changes that might encourage new approaches to preventing disease—for example, by moving away from activity-based payment systems and introducing an ICS performance framework that may include population-level outcome metrics. The logic is that these policy changes may incentivise ICS members to work together to prevent disease, improve health, and reduce service spend. However, international evidence on the impact of closer integration of organisational governance structures and budgets does not, so far, provide much guidance on how such policies will improve population health.^24 30–34 45^

## What can be learned from the 2016 STP plans?

Given the potential limitations of the Long Term Plan’s approach to disease prevention, we analysed the 2016 STP plans to understand how they intended to prevent disease and to see what can be learnt in advance of the publication of the 2019 ICS and STP plans.

We analysed the 44 STP plans, coding content related to disease prevention and population health. Our analysis is framed using the 2015 NHS England planning guidance for implementing the Five Year Forward View and Public Health England’s (PHE) menu of preventative interventions.^9 46^ NHS England’s planning guidance set out a series of questions on prevention for STPs to consider when developing their plans, and in 2016 (shortly after plans were submitted), PHE produced a menu of ‘evidence-based, preventative public health interventions that can help improve the health of the population and reduce health and care service demand in the short to medium term’. (see Box 1).

Box 1 **Content of national guidance documents for 2016 sustainability and transformation partnership (STP) plans related to disease prevention^9 46^**

National Health Service (NHS) England’s ‘Delivering the Forward View, NHS planning guidance 2016/17 – 2020/21’ was published in December 2015 and set out the requirement for local NHS organisations to produce a 5-year Sustainability and Transformation Plan.^9^ The report included the following questions for planners to consider with regards to preventing ill health and improving population health:

- How will you assess and address your most important and highest cost preventable causes of ill health to reduce healthcare demand and tackle health inequalities working closely with local government?
- How rapidly could you achieve full local implementation of the national Diabetes Prevention Programme? Why should Public Health England (PHE) and NHS England prioritise your geographical area (eg, with national funding to support the programme)?
- What action will you take to address obesity, including childhood obesity?
- How are NHS and other employers in your area going to improve the health of their own workforce, for example, by participating in the national roll out the Healthy NHS programme?
- How will you deliver a transformation in cancer prevention, diagnosis, treatment and aftercare in line with the cancer task force report?

PHE developed a guidance document for STPs: ‘Local health and care planning - menu of preventative interventions’.^46^ The report was published shortly after the 2016 plans were submitted and provides local STP leaders with a list of preventative interventions that could both ‘improve the health of the population and reduce health and care service demand in the short to medium term’. PHE advised that the list does not cover the full breadth of interventions that can help prevent ill health, and any plans should be informed by local knowledge.

Among the 14 topic areas covered by the guidance, six recommended interventions were estimated to improve health and well-being, and save money to the health and/or care sector within 5 years:

- Alcohol: identification and brief advice in primary care.
- Alcohol: alcohol care teams in secondary care.
- Tobacco: screening, advice, and referral in secondary care.
- Hypertension: improved management of hypertension in primary care.
- Contraception: increase uptake of long-acting reversible contraceptives in general practice, maternity and abortion pathways.
- Falls: implement a fracture liaison service in secondary care.

Our findings suggest that STPs could do more to improve preventive care planning and service delivery (see online supplementary table 1 for detailed results and how plans compare with national guidance). Although STPs were asked by NHS England to include plans for a ‘radical upgrade’ in prevention and to describe how the most important preventable causes of ill health would be assessed and addressed, fewer than half of plans included detail on how they will work with their local government public health team to identify and meet these needs. There was also incomplete coverage across plans about how STPs intended to meet other NHS England prevention priorities, such as implementing the National Diabetes Prevention Programme, improving the health of their workforce, transforming cancer prevention, and tackling childhood obesity.

10.1136/ihj-2019-000013.supp1 Supplementary data

Prevention strategies commonly focused on individual-level programmes targeting behaviour change rather than on more upstream or population-level approaches that might be more likely to improve overall population health and reduce inequalities,^47^ and although 37 of the 44 STP plans included some mention of either how prevention activities would be funded or quantified potential savings, only six included detail on how local costs and savings were estimated. This lack of detail makes it difficult to know how these estimates were calculated and is consistent with previous analyses of the plans showing unrealistic projected reductions in service use and efficiency savings.^19 48^ Our findings are limited, however, by only analysing what STP plans said rather than analysing what STPs subsequently did, and there remains the possibility that prevention plans existed elsewhere within some STP areas.

## What more can local health and care systems do to prevent disease?

ICSs and STPs represent an opportunity to implement more systemic approaches to prevent disease and improve population health. To do this, we hope that 2019 plans will go beyond the 2016 STP plans and fill some of the gaps in the Long Term Plan on social determinants.

First, local government, along with volunteer, community, and social enterprise (VCSE) organisations, need to be core partners in plans’ development and priority setting. Population-level prevention interventions and programmes aimed at addressing social determinants of health—including social prescribing—rely on non-NHS organisations. Their involvement will help identify gaps in services and develop strategies for how these might be overcome. Achieving partnership working between health, social care, and community sectors to improve health is difficult^37 38^ and can be compounded by VCSEs having to operate in competitive funding environments where each organisation has different aims and incentives.^43^ VCSE coordination and engagement with local health and care organisations is increasingly being mediated through local VCSE infrastructure organisations that can collectively represent local VCSE interests.^49^ Insight from local government and community groups is crucial for ICSs and STPs to understand what is likely to be achievable and—more importantly—what is not.

Second, despite ongoing system-wide financial pressures, there is much that NHS provider organisations can do to prevent disease and improve local population health using their existing resources and assets. For example, NHS organisations could use their role as major local employers, purchasers and partners to other organisations to strengthen the social and economic conditions in their community.^50 51^ Frameworks developed for health systems in other countries have also described the patient and population-level interventions that healthcare systems could pursue to identify and respond to patients’ social circumstances.^52^ To support NHS provider organisations in doing this, the content and number of national performance incentives and outcome frameworks needs to be systematically reviewed to align more closely with prevention and population health goals.^19 53^

Third, the anticipated impact of proposed interventions on health, service use, and costs should be more realistically quantified. STP plans from 2016 were often wildly optimistic about the impact of interventions on service use.^19^ More recent data suggest that, in some contexts, service integration may increase costs through identifying unmet patient needs.^26^ The development of linked local health and care records and the NHS’s ambition to improve analytical capability will help ICSs and STPs to better understand the needs of their populations, and to evaluate and learn from the potential impact of proposed interventions.^54^ This will also help develop the evidence for how organisational and service integration affects disease prevention and population health.

The NHS can only do so much to address social determinants of health and improve disease prevention. Even with more effective partnership working at a local level, the health of local populations is fundamentally shaped by macro-level political and economic factors, such as the amount and distribution of spending on welfare and public services.^55^ Any local efforts by the NHS and its partners must therefore be supported by a coordinated approach to prevention across central government, including increasing local government funding after a decade of austerity, and reversing cuts to public health budgets.^56^ Without such broader cross-government support, STPs and ICSs will be set up to fail in their ambition to improve population health. The NHS Long Term Plan and prevention green paper are unlikely to be enough,^57^ and only when we know the content of the UK government’s spending plans will we really know how seriously we should all take the prevention rhetoric.
