# Supplementary material for: Integrated health and care systems in England: can they help prevent disease?
Source: Integr Healthc J. 2020 Feb 26;2(1):e000013. doi: 10.1136/ihj-2019-000013 (PMC10327462; doi:10.1136/ihj-2019-000013)
Supplement: Supplementary data [file ihj-2019-000013supp001.pdf]

Supplementary table. Findings from our analysis of the prevention content of 2016 Sustainability and Transformation Plans, and how they relate to 2016 national guidance [1,2]

Finding	Example	Consistency with evidence or national guidance
Forty-one of the 44 plans include a population health or prevention strategy, two of the remaining three have a section on prevention or population health.	Surrey Heartlands is an example of an STP plan that includes a prevention strategy and weaves prevention throughout the plan's other sections.	NHS England's planning guidance states that STPs must integrate with local government services and include plans for a 'radical upgrade' in prevention.
Eighteen plans described how they would work with their local government public health teams to develop their preventive care strategies, for example, through using local needs assessments to plan services. A further 12 plans included limited information about how the local government public health team is part of the STP.	Northumberland, Tyne and Wear STP's plan includes public health specialists when identifying prevention priorities and solutions throughout.	NHS England's planning guidance asks planners: "How will you assess and address your most important and highest cost preventable causes of ill health, to reduce healthcare demand and tackle health inequalities working closely with local government?" The NHS Long Term Plan Implementation Framework also recommends that ICSs and STPs engage with the local Directors of Public Health to develop prevention plans.[3]
Twenty-four of the 44 plans include implementing or expanding the National Diabetes Prevention Programme.	Cheshire and Merseyside STP anticipate that expanding the Diabetes Prevention Programme to cover 5,000 patients could lead to an annual saving the local health system of over £500k per year by 2021.	The University of Sheffield NHS Diabetes Prevention Programme Return on Investment Tool is estimated to save health and social care around £90 per person over 15 years and is a priority for both NHS England and PHE.
A systematic approach to tackling childhood obesity is only included in five plans, and when included, detail is generally lacking.	Durham, Darlington, Teeside, Hambleton, Richmondshire and Whitby STP includes a proposal to develop a four-year comprehensive childhood obesity plan.	How planners will address childhood obesity is specifically asked about in NHS England's planning guidance, and a range of evidence-based interventions are suggested in PHE's

		menu of interventions.
Twenty-one plans include proposals to support workplace wellbeing, with the Healthy NHS Programme only being mentioned in one plan.	South East London STP states their plan to develop a Healthier Workforce Strategy including committing to the roll-out of the Healthy NHS Programme.	NHS England's planning guidance asks, "How are NHS and other employers in your area going to improve the health of their own workforce – for example by participating in the national roll out the Healthy NHS programme?" and PHE's menu of interventions includes a section on evidence-based interventions to improve workplace health and wellbeing.
Cancer was often included as a priority, with proposals to improve its early identification included in 11 plans.	In South Yorkshire and Bassetlaw, planners propose improving early cancer detection through primary care education and support, using prediction software, reducing variation in screening uptake, and linking with national awareness campaigns.	NHS England's planning guidance asks planners to consider how they will deliver a transformation in cancer prevention.
Of the 44 plans, 15 do not mention any of the six PHE recommendations estimated to improve health and save money within five years.	Sixteen plans included the introduction or expansion of alcohol services focusing on one or both of identification and brief advice in primary care, and alcohol care teams in secondary care.	These are the PHE six recommended interventions that are estimated both to improve health and save money within five years of implementation.
	The introduction of tobacco cessation services into secondary care was mentioned by 18 plans	
	Improving the management of hypertension in primary care, increasing uptake of long-acting reversible contraception, and implementation of a fracture liaison service was only explicitly mentioned in four, five and four plans respectively.	
Training health care workers in Making Every Contact Count (MECC) – providing	Bristol, North Somerset and South Gloucestershire STP plan includes a detailed	This approach to behaviour change is suggested through PHE's menu of

brief advice on healthy lifestyles – featured in 24 plans.	MECC project covering diet, smoking, physical activity, alcohol, and mental wellbeing. It includes having MECC coordinators, MECC champions among senior leaders, and staff training programmes.	preventative interventions and forms part of the NHS Standard Contract which states that providers need to develop and maintain an organisation plan for MECC.
Prevention or early identification of mental illness was a common focus, with adult mental health prevention mentioned in 16 plans, and perinatal mental health is specifically highlighted in six plans.	Cambridgeshire STP describe how they intend to take a whole-system life-course approach to mental health and wellbeing, investing in initiatives to prevent, identify, and treat mental illness in children, among families, and in adults.	Mental health is highlighted as a priority in NHS England's planning guidance, it asks providers how they will improve mental health services. PHE's menu of interventions also includes a section on mental health, emphasising the strong evidence base underlying the provision of a comprehensive perinatal mental health service.
Population-level approaches to disease prevention were mentioned infrequently and when included, were often non-specific.	For example, four plans suggest using licensing laws to reduce harm from alcohol; Cornwall and the Isles of Scilly STP plan includes proposals to increase active travel.	Population-level plans often require lower levels of individual agency and as such, may be more likely to be effective and equitable than individual-level plans.[4] Increasing active travel is one of several included in PHE's menu of interventions.
Thirty-two STP plans included content related to social determinants of health – this ranged from simply mentioning the importance of the social determinants of health, through to describing detailed programmes of work. The most frequently mentioned topics were employment (nine plans), housing (eight plans), and using social prescribing (16 plans).	Nottinghamshire's STP plan includes a detailed section on how they plan to improve housing and the local built environment, including key outcomes and milestones for the local health and care system.	The majority of what influences our health and wellbeing lies outside of healthcare systems and is determined by our environmental and economic circumstances. Improving housing quality and employment opportunities are likely to have important physical and mental health benefits (as mentioned in PHE's menu of interventions). However, the evidence underlying social prescribing is less well developed.[5]

Notes: We analysed all 2016 STP plans that were submitted to NHS England. This included 43 STP plans and Greater Manchester's 2015 plan for reforming health and social care following its devolution agreement. One STP plan referenced a separate prevention strategy document that we were unable to access (we count this in the 41 STP plans with a prevention or population strategy). Plans were coded for all content relating to disease prevention (defined as information relating to the primary or secondary prevention of disease) and population health (information relating to addressing the mental and physical health and wellbeing of a defined population for which the STP is accountable, including efforts to reduce inequalities within that population). Themes were identified and analysed covering topics such as interventions, organisational determinants, and strategy and planning.

References

1 NHS England. Delivering the Forward View: NHS planning guidance 2016/17 – 2020/21. London: 2015.

2 Public Health England. Local health and care planning: menu of preventative interventions. London: 2016. https://assets.publishing.service.gov.uk/government/uploads/system/uploads/attachment_data/file/683016/Local_health_and_care_planning_menu_of_preventative_interventions_DM_NICE_amends_14.02.18__2_.pdf

3 NHS England. NHS Long Term Plan Implementation Framework. London: 2019.

4 Adams J, Mytton O, White M, *et al.* Why Are Some Population Interventions for Diet and Obesity More Equitable and Effective Than Others? The Role of Individual Agency. *PLoS Med* 2016;**13**:e1001990. doi:10.1371/journal.pmed.1001990

5 Bickerdike L, Booth A, Wilson PM, *et al.* Social prescribing: less rhetoric and more reality. A systematic review of the evidence. *BMJ Open* 2017;**7**:e013384. doi:10.1136/bmjopen-2016-013384
